# Pharmacokinetics, Tissue Distribution, and Human Serum Albumin Binding Properties of Delicaflavone, a Novel Anti-Tumor Candidate

**DOI:** 10.3389/fphar.2021.761884

**Published:** 2021-11-17

**Authors:** Bing Chen, Hongbin Luo, Weiying Chen, Qishu Huang, Kaifan Zheng, Dafen Xu, Shaoguang Li, Ailin Liu, Liying Huang, Yanjie Zheng, Xinhua Lin, Hong Yao

**Affiliations:** ^1^ Key Laboratory of Nanomedical Technology (Education Department of Fujian Province), School of Pharmacy, Nano Medical Technology Research Institute, Fujian Medical University, Fuzhou, China; ^2^ Department of Pharmaceutical Analysis, School of Pharmacy, Fujian Medical University, Fuzhou, China; ^3^ Department of Orthopedic, The First Affiliated Hospital, Fujian Medical University, Fuzhou, China; ^4^ Department of Pharmacy, Xiamen Humanity Hospital, Fujian Medical University, Xiamen, China; ^5^ Fujian Key Laboratory of Drug Target Discovery and Structural and Functional Research, Fujian Medical University, Fuzhou, China

**Keywords:** delicaflavone, pharmacokinetics, tissue distribution, human serum albumin, plasma binding property, antitumor candidate 2

## Abstract

Delicaflavone (DF), a natural active ingredient from *Selaginella doederleinii* Hieron, has been reported to have favorable anticancer effects and is thus considered a potential anticancer agent. However, its pharmacokinetics and plasma protein binding properties remain unknown. Here, we investigated the pharmacokinetic profile of DF in rats using a validated HPLC-MS/MS methods, as well as its human serum albumin (HSA) binding properties through multi-spectroscopic and *in silico* methods. The results showed that DF was rapidly eliminated and had a widespread tissue distribution after intravenous administration. DF showed linear dynamics in the dose range of 30–60 mg/kg and poor oral bioavailability. The major distribution tissues of DF were the liver, lungs, and kidneys. Ultraviolet and fluorescence spectroscopy and molecular docking demonstrated that DF had a static quenching effect on HSA, with one binding site, and relatively strong binding constants. Thermodynamic analysis of the binding data revealed that hydrogen bonding and van der Waals interactions played major roles in binding. The results of this study further our understanding of the pharmacokinetic and plasma protein binding properties of the potential anticancer agent DF and shed light on pharmacological strategies that may be useful for the development of novel cancer therapeutics.

## 1 Introduction

Delicaflavone (DF), a rarely occurring biflavonoid, is an active ingredient isolated from *Selaginella doederleinii* Hieron, a traditional Chinese medicine used for health promotion and treatment of various cancers (Liu et al., 2011; Li et al., 2020). Modern pharmacological studies have demonstrated that DF induces reactive oxygen species (ROS)-mediated apoptosis and cell cycle arrest via the PI3K/AKT/mTOR and Ras/MEK/Erk signaling pathways, which results in autophagic cell death via the Akt/mTOR/p70S6K signaling pathway (Sui et al., 2017; Yao et al., 2020). In summary, the existing research indicates that DF possesses favorable anti-tumor potential and excellent safety profiles, and deserves further research and development as a novel anti-tumor candidate either alone or in combination with other drugs (Sui et al., 2016; Chen et al., 2019; Yao et al., 2019). However, the pharmacokinetic and plasma binding properties of DF have not been fully elucidated.

Pharmacokinetic assessments are a prerequisite for elucidating the mechanisms of DF, which is essential for lead identification and optimization in drug discovery and preclinical development, as well as for dosage regimen design and drug formulation (Li et al., 2019; Chen et al., 2020; Kamble et al., 2021). Several studies have evaluated the pharmacokinetic characteristics of biflavonoids in *S. doederleinii* (Liao et al., 2015). However, there is not enough information on the *in vivo* process profiles of individual DF (Yao et al., 2017). Thus, it is important to propose a systematic and comprehensive study on the pharmacokinetics of DF *in vivo* to further understand its pharmacological effects and to develop its clinical application (Su et al., 2019; Zhu et al., 2021).

Serum albumin is the most abundant protein and the most important transport protein in the blood circulatory system and plays a critical role in pharmacokinetics and pharmacodynamics (Volpp and Holzgrabe, 2019). Plasma protein binding rate is an important parameter from a pharmacokinetic and pharmacodynamics perspective and has become a widely accepted parameter during the development of new drugs (Nation et al., 2018). However, previous descriptions on the pharmacokinetic behavior of DF were not comprehensive, and the plasma protein binding property to human serum albumin (HSA) has not been reported before (Wang et al., 2021).

In this study, a reliable and highly sensitive HPLC-ESI-MS/MS method was developed and validated for quantitative of delicaflavone. The comprehensive pharmacokinetics and tissue distributions of individual DF in rats and the binding properties of DF are presented for the first time. Our work presents a comprehensive description of the pharmacokinetic behaviors of DF in rats and the association between DF and HSA, which may provide a useful methodological reference for future assays designed to evaluate its human applications.

## 2 Materials and Methods

### 2.1 Chemicals and Reagents

DF (purity ≥98.0%) was supplied by Fujian Medical University (Fuzhou, China). Amentoflavone (purity ≥98.0%, internal standard, IS) was obtained from Shanghai Winherb Medical Technology Co., Ltd. (Shanghai, China). Free-fatty acid HSA was purchased from Sigma-Aldrich (St. Louis, MO, United States), and ultrafiltration disk membranes (molecular weight cut-off 10 kDa) were purchased from Merck Millipore (Darmstadt, Germany). HPLC-grade acetonitrile and methanol used in the study were purchased from Merck (Darmstadt, Germany), and the HPLC-grade glacial acetic acid was obtained from Aladdin (Shanghai, China). Water was purified using a Milli-Q system (Millipore, Bedford, MA, United States). All other chemicals and reagents used in this study were of analytical grade.

### 2.2 Animals

Sprague-Dawley rats (200 ± 20 g) were supplied by the Laboratory Animal Center of the Fujian Medical University. All the animals were housed in an environmentally controlled breeding room with an ambient temperature of 25 ± 2°C and a relative humidity of 55 ± 5% with a 12 h light/dark cycle and were observed for 1 week before the experiment. The rats were fed chow and water *ad libitum*, and fasted for 12 h before drug administration, but were allowed free access to water. All animal studies were conducted in accordance with the Guidelines for the Care and Use of Laboratory Animals approved by the Animal Ethics Committee of Fujian Medical University.

### 2.3 Preparation of Stock Solutions, Calibration Standards, and Quality Control Samples

Standard stock solutions of DF and IS were dissolved in ethanol at concentrations of 200 and 100 μg/ml, respectively. The working solutions of DF for the calibration standards were prepared daily by diluting the stock solution with ethanol. The stock solution of IS was prepared and further diluted to obtain a working solution of 1 μg/ml. Then, the mixed stock solution was diluted with the IS working solution to provide a series of working standard solutions for the calibration curve. All the solutions were stored at 4°C until use.

Calibration standards of DF were prepared by spiking 10 μl standard working solutions to 100 μl blank plasma and tissue homogenates, and then samples were vortexed for 3 min to obtain a series of concentrations of: 1, 10, 50, 100, 200, 500, 1,000, 2,000, 5,000, and 10,000 ng/ml in the blank plasma; and 10, 20, 50, 100, 200, 500, 1,000, 2,000, and 4,000 ng/ml in the tissue homogenates. QC samples were prepared in the same manner at three different concentrations (3, 750, and 8,000 ng/ml for plasma; and 30, 300, and 3,000 ng/ml for tissue homogenates).

### 2.4 Pharmacokinetic and Tissue Distribution Studies

Twenty-four Sprague-Dawley rats (male, weight 200 ± 20 g) were randomly divided into four groups (*n* = 6) and used for the pharmacokinetic and bioavailability studies. DF was suspended in ethanol, polyethylene glycol 400 (PEG 400), and physiological saline (15:30:55, v/v/v) and was administered to the rats. Approximately 300 µl of blood samples was collected from the tail vein of each rat into heparinized tubes at 0.083, 0.25, 0.5, 0.75, 1, 1.5, 2, 4, 8, 12, and 24 h after a single oral administration (*i.g.*) (30, 45, and 60 mg/kg) or at 0.042, 0.083, 0.167, 0.25, 0.5, 0.75, 1, 1.5, 2, 4, 8, 12, and 24 h after a single *i.v.* injection (4 mg/kg) (Sui et al., 2017; Yao et al., 2019). Plasma was immediately isolated from the blood samples by centrifugation at 3,000 × g for 10 min, and then stored at −80°C until analysis. The absolute oral bioavailability (F) was calculated using the following formula:

$$F=\;\frac{\left(AUC_{i.\;g.}\times dose_{i.v.}\right)}{\left(AUC_{i.v.}\times dose_{i.g.}\right)}\times 100.$$ (1)

Thirty Sprague-Dawley rats (15 females, 15 males, weight 200 ± 20 g) were randomly divided into five groups (*n* = 6) for the tissue distribution study. At each time point (0.083, 0.25, 0.5, 1, and 2 h) following administration of a single *i.v.* dose (4 mg/kg) of DF, a group of animals (three females and three males for each treatment time) were sacrificed. Tissues (including the heart, liver, spleens, lungs, kidneys, brain, testis, ovary, and muscle) were immediately harvested and thoroughly rinsed in ice-cold physiological saline to remove the superficial blood and other contents. All tissue samples were processed using the methods described in the following section, immersed immediately in an ice bath at the end of each collection interval, and then stored at −80°C until analysis.

### 2.5 Determination of Drug Concentration

#### 2.5.1 Sample Preparation

The plasma and tissue homogenate samples were obtained as follows: 100 μl biological samples were spiked with an aliquot of 10 μl IS working solution, vortex-mixed with 300 μl of methanol for 2.5 min, and centrifuged at 15,000 g for 10 min at 4°C to precipitate proteins. Subsequently, 5 μl of the supernatant was injected into the HPLC-MS/MS system for analysis.

#### 2.5.2 Instrument and Analytical Conditions

The samples were analyzed using a Shimadzu LC/MS-8040 system (Shimadzu, Kyoto, Japan) connected to a Shimadzu LC-20AD HPLC system (Shimadzu, Kyoto, Japan). An Ultimate^®^ XB-C18 (50 × 4.6 mm, 3.5 μm; Welch Materials, Inc., Ellicott, MD, United States) column was used for separation; the column temperature was 30°C, the flow rate was 0.4 ml/min, and the injection volume was 5 μl. The mobile phase was composed of a mixture of 0.5% glacial acetic acid water (A) and acetonitrile (B), and the separation was achieved within 5 min using isocratic elution at a ratio of 42% A and 58% B.

**FIGURE 1:**
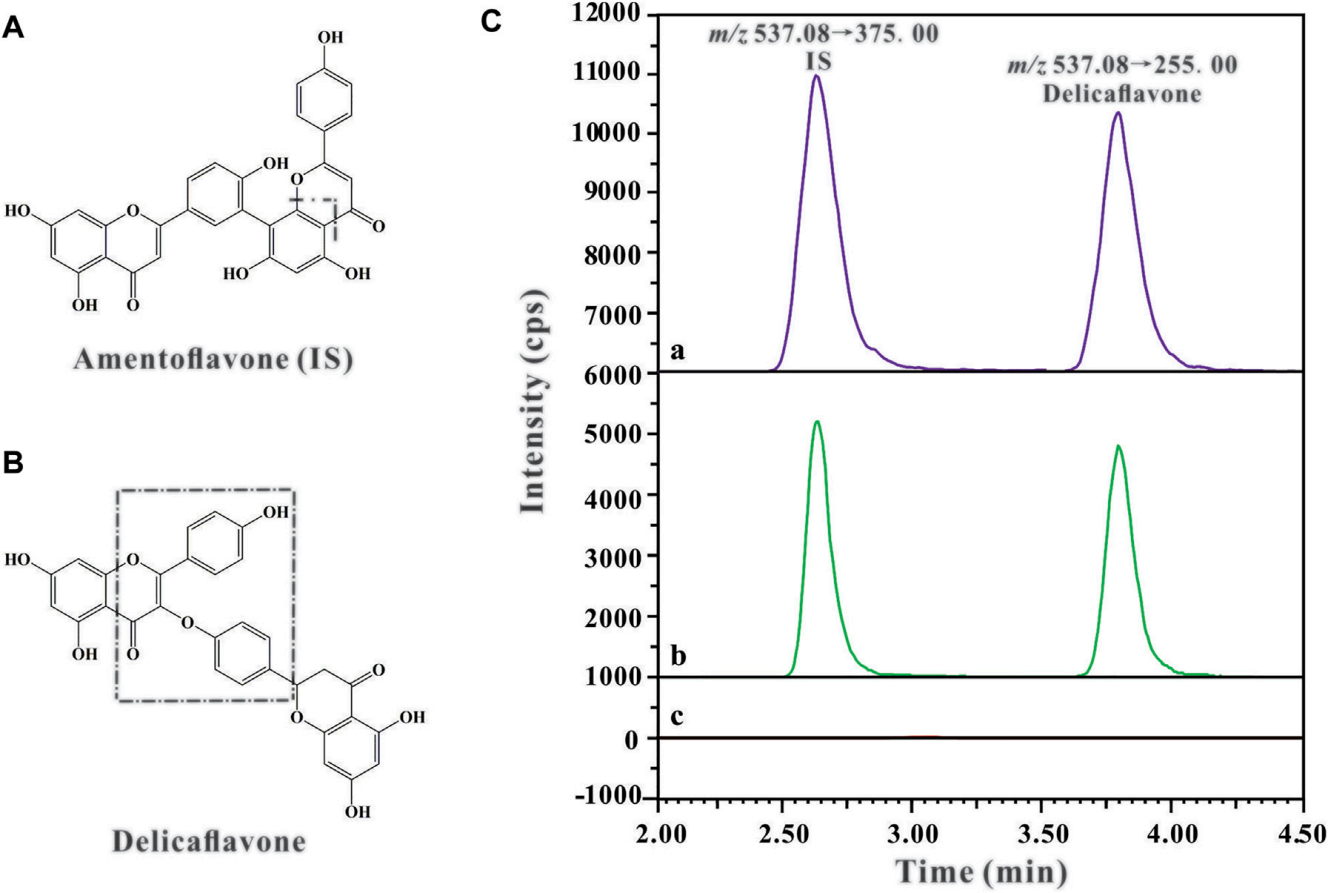
The chemical structures and fragmentation schemes for **(A)** Amentoflavone (IS), **(B)** Delicaflavone, and **(C)** Representative chromatograms of IS (100 ng/ml) and DF in rat plasma sample: **(A)** Plasma sample collected from the rat 2 h after *i.v.* administration of DF at a dose of 4 mg/kg; **(B)** Blank plasma spiked with DF and IS; **(C)** Blank plasma sample.

Triple-quadrupole tandem mass spectrometric detection (Shimadzu LC/MS-8040 system; Shimadzu, Kyoto, Japan) was coupled with an electrospray ionization interface. The detection and quantification of the analyte were performed using multiple reaction monitoring mode of the transitions *m/z* 537.20→255.20 for DF and *m/z* 537.20→375.00 for IS in the negative mode, based on our previous method (Chen et al., 2018). The chemical structures and fragmentation schemes of DF and IS are shown in Figures 1A,B. The optimized MS parameters were as follows: ion spray voltage, 6.0 kV; DL temperature, 250°C; heat block temperature, 400°C; nebulizer gas flow, 3 L/min; drying gas flow, 12 L/min; and the collision energies (CE) for DF and amentoflavone were 35 and 48 eV, respectively. Data acquisition and processing were performed using LabSolutions LCMS Ver. 5.5 software.

#### 2.5.3 Method Validation

The developed HPLC-MS/MS method was validated in accordance with the Food and Drug Administration guidelines for industry on bioanalytical method validation procedures. The indicators of evaluation included specificity, linearity, inter- and intra-day accuracy and precision, extraction recovery, LLOQ, and stability of DF in the blank plasma and tissue homogenates.

### 2.6 Plasma Protein Binding and Blood-Plasma Partitioning

The ultrafiltration method was used to determine the extent of plasma protein binding *in vitro*. Briefly, an aliquot of 10 μl DF working solution (3.5, 35, and 350 μg/ml) was spiked into 700 μl blank plasma to give final concentrations of 50, 500, and 5,000 ng/ml and incubated for 1 h at 37°C. After an incubation period, an aliquot of 100 μl plasma sample was removed for drug concentration analysis, while the other 600 μl was transferred to a 10 kD cut-off ultrafiltration device (Darmstadt, Germany) and centrifuged at 2,000 × g for 2 h at 37°C. An aliquot of 100 μl of the centrifuged plasma sample was analyzed for the free drug concentration using the LC-MS/MS method. The percentage of protein binding was calculated using the following formula (Correa-Basurto et al., 2019):

$$Protein\;binding\;ratio\;\left(\%\right)=\left[1-\left(\frac{drug\;ultrafiltrate}{total\;drug}\right)\right]\times 100\%.$$ (2)

The blood/plasma concentration ratio (BP) of DF was determined by dividing 100 ng/ml and 1,000 ng/ml, respectively. DF was added to whole blood to obtain a final concentration and then these samples were incubated at 37°C for 30 min. Plasma was immediately isolated from the blood samples and processed using the methods described in sample preparation, and the concentration of DF was determined from a calibration curve that was prepared with blank plasma using the LC-MS/MS method.

### 2.7 Binding Studies on HSA

#### 2.7.1 UV-Visible Spectroscopy Measurements

Interactions of the ligand (DF) with HSA were studied using UV-visible spectroscopy. The UV-visible absorption spectra of HSA (15 μM) in the absence or presence of various concentrations of DF (5.00, 7.50, 10.00, and 12.50 μM) were recorded from 240 to 470 nm using a spectrophotometer with 1.0 cm optical path quartz cuvette.

#### 2.7.2 Fluorescence Measurements

##### 2.7.2.1 Intrinsic Fluorescence Measurements

The intrinsic fluorescence spectra of HSA (8 μM) in phosphate buffer (pH 7.4) were incubated in the absence and presence of different concentrations of DF (5, 10, 15, 20, 25, 30, 35, 40, 45, and 50 μM) and were recorded at 37°C. An excitation wavelength of 295 nm was used throughout the experiments to avoid interference from tryptophan and/or tyrosine residues. To avoid the inner filter effect, the fluorescence data were corrected using the following formula:

$$F_{corr}=\;F_{obs}\times antilog\left[\frac{\left(A_{em}+A_{ex}\right)}{2}\right],$$ (3)

where F_corr_ and F_obs_ represent the corrected and observed fluorescence intensities respectively, and A_em_ and A_ex_ are the absorbances of the test sample at the excitation and emission wavelengths, respectively (Balaei and Ghobadi, 2019).

##### 2.7.2.2 Determination of the Fluorescence Quenching Mechanism

Fluorescence quenching experiments were performed by adding various concentrations of DF (5, 10, 15, 20, 25, 30, 35, 40, 45, and 50 μM) to HSA (8 μM) at different temperatures (298, 303, 308, and 313 K). The emission spectra of DF-HSA complex were recorded in the range of 305–450 nm and the decrement in fluorescence intensity at the maximum of emission was determined according to the Stern-Volmer equation:

$$\frac{F_{0}}{F}=1+K_{SV}\left[Q\right]=1+\;k_{q}\tau_{0}\left[Q\right],$$ (4)

where F_0_/F are the fluorescence intensities in the absence and presence of the quencher (DF), respectively, [Q] is the molar concentration of the DF (quencher), K_SV_ is the dynamic quenching constant, K_q_ is the apparent bimolecular quenching rate constant, and τ_0_ is the fluorophore lifetime without DF (quencher), which has a value of 10^−8^ s (Yue et al., 2017).

##### 2.7.2.3 Determination of the Binding Parameters and the Thermodynamic Parameters of the Binding Process

The binding of small molecules to a set of equivalent sites on a macromolecule can be calculated using the modified Stern-Volmer equation:

$$\log\left\{\frac{F_{0}-F}{F}\right\}=\log K_{b}+n\log\left[Q\right]\;.$$ (5)

The thermodynamic parameters of the binding event can be used to elucidate the binding modes between ligands and macromolecules. Therefore, the thermodynamic parameters of the binding constant were investigated at four different temperatures (298, 303, 308, and 313 K). The entropy change (ΔS°) and the enthalpy change (ΔH°) were obtained from the linear van’t Hoff plot to characterise the forces acting between DF and HSA:

$$\ln K_{b}=-\left(\frac{\text{Δ}H{}^{\circ}}{RT}\right)+\left(\frac{\text{Δ}S{}^{\circ}}{R}\right),$$ (6)

where K_b_ is the binding constant, R is the universal gas constant, T is the absolute temperature, and n is the number of binding sites per macromolecule at the corresponding temperature. Assuming that ΔH° is nearly constant in the studied temperature range, the free energy change (ΔG°) can be analyzed from the Gibbs equation:

ΔG°= ΔH°−ΔS°. (7)

### 2.8 Molecular Modeling Study

Molecular docking simulation was carried out using Sybyl-x 1.3 (St. Louis, MO) to study the interaction between DF and HSA. The three-dimensional (3D) crystal structure of the HSA protein model (PDB ID: 1BM0) was downloaded from the RCSB Protein Data Bank (http://www.rcsb.org/). Water molecules were removed from the crystal structure, while the hydrogen atoms, N-termini and C-termini state were fixed during protein preparation. Ligands were drawn and subjected to structural minimization using Compute Sybyl-x 1.3. The Surflex-Dock (SFXC) docking mode was used to assess the possible conformation of the drug-protein complexes, and Surflex-Dock scores (total scores) represented the binding affinities. Drug-protein complexes were analyzed using the PyMOL program.

### 2.9 Data Analysis

Data acquisition and processing were conducted using LabSolutions LCMS Ver.5.5 software. The concentrations of DF in the plasma and tissues were determined using the calibration curve of each analysis batch. The pharmacokinetic parameters of DF in plasma were calculated using a non-compartmental approach with DAS 3.0 Version (Mathematical Pharmacology Professional Committee of China, Shanghai, China). The C_max_ and T_max_ were obtained directly from the experimental process.

All data are expressed as the mean ± SD. Independent t-tests were performed to assess the differences between two groups. Statistical significance was set at *p* < 0.05.

## 3 Results

### 3.1 Method Validation

#### 3.1.1 Specificity

Representative chromatograms of blank rat plasma, blank rat plasma spiked with amentoflavone (IS) and DF, and rat plasma samples 2 h after *i.v.* administration of DF are shown in Figure 1C. It can be observed that the retention times of DF and IS were around 2.55 and 3.65 min, respectively. The results showed excellent method selectivity; that is, the endogenous components from the biological samples (plasma and tissue homogenate) did not interfere with the retention times of DF and IS.

#### 3.1.2 Linearity and LLOQ

The linearity of DF in plasma was obtained over a range of 1–10,000 ng/ml using a 1/X^2^ weighting. Linearity of DF in tissue homogenate was obtained over a range of 10–4,000 ng/ml using a 1/X^2^ weighting (Table 1). The correlation coefficients of the calibration curves listed in the table indicate that DF had excellent linearity in the rat bio-samples (R^2^ = 0.999). The lower limit of quantification (LLOQ) of DF was set at 1 ng/ml for plasma and 10 ng/ml for tissues.

**TABLE 1:** Precision and accuracy of the assay method for DF in biological sample (*n* = 5).

Biological sample	Linear range	Regression equation (w = 1/x^2^)	Precision (RSD,%)			Accuracy	
	(ng/ml)		Conc. (ng/ml)	Intraday	Interday	Conc. (ng/ml)	RE (%)
Plasma	1–10,000	y = 0.0023x+0.0029 (R^2^ = 0.999)	3	5.21	5.49	3	4.57
			750	2.12	2.07	750	−2.05
			8,000	0.98	1.58	8,000	−1.76
Heart	10–4,000	y = 0.0042x+0.0003 (R^2^ = 0.999)	30	3.25	4.71	30	−2.33
			300	3.13	4.66	300	−2.30
			3,000	1.17	3.48	3,000	1.30
Liver	10–4,000	y = 0.0052x-0.0030 (R^2^ = 0.999)	30	5.86	5.25	30	−6.06
			300	0.97	5.14	300	−4.98
			3,000	2.21	2.04	3,000	−2.02
Spleen	10–4,000	y = 0.0043x+0.0127 (R^2^ = 0.999)	30	1.51	4.14	30	−4.85
			300	2.14	5.13	300	3.64
			3,000	0.54	3.64	3,000	−4.24
Lung	10–4,000	y = 0.0028x+0.0065 (R^2^ = 0.999)	30	1.93	1.73	30	−4.93
			300	1.56	2.39	300	3.72
			3,000	0.48	1.16	3,000	−1.93
Kindey	10–4,000	y = 0.0049x+0.0005 (R^2^ = 0.999)	30	2.38	3.91	30	1.91
			300	2.05	5.39	300	−2.74
			3,000	5.72	5.03	3,000	−8.01
Brain	10–4,000	y = 0.0028x+0.0065 (R^2^ = 0.999)	30	6.27	4.38	30	5.13
			300	4.82	5.25	300	−4.54
			3,000	1.98	3.22	3,000	1.27
Testis	10–4,000	y = 0.0013x+0.0090 (R^2^ = 0.999)	30	1.69	2.86	30	−3.48
			300	2.71	2.74	300	−2.56
			3,000	2.79	1.21	3,000	3.45
Ovary	10–4,000	y = 0.0033x+0.0026 (R^2^ = 0.999)	30	5.23	6.02	30	−4.53
			300	3.54	3.94	300	−4.01
			3,000	2.93	3.38	3,000	2.32
Muscle	10–4,000	y = 0.0019x+0.0077 (R^2^ = 0.999)	30	6.12	5.94	30	−3.27
			300	4.53	5.21	300	−1.94
			3,000	3.65	3.46	3,000	−2.31

Conc., concentration. RSD, relative S.D. (calculated from S.D., divided by mean and multiplied by 100).

#### 3.1.3 Accuracy and Precision

The accuracy and precision of the method for quantification of DF were evaluated by analyzing the RE and RSD of quality control (QC) samples (LQC, MQC, and HQC) in the different biological samples (Table 1). The intra- and inter-day precisions (RSD) for DF in the QCs were less than 6.27%, and the accuracy (RE) ranged from −8.01 to 5.13%. The results demonstrate that the proposed method has good accuracy and precision.

#### 3.1.4 Extraction Recovery and Matrix Effect

The extraction recoveries of DF and IS at the three levels of QC samples ranged from 86.96 to 99.67% with no significant variation. The matrix effect of the analytes was 99.35 ± 8.05% (Table 2). These results revealed that the recoveries of DF in the different matrices were within an acceptable range, and there were no notable endogenous interferences for the detection of DF in the rat bio-samples.

**TABLE 2:** Matrix effect and extraction recovery of DF in rat plasma and tissues homogenate (*n* = 5).

Biological samples	Spiked concentration	Matrix effect		Extraction recovery	
	(ng/ml)	Mean ± SD (%)	RSD%	Mean ± SD (%)	RSD%
Plasma	3	89.26 ± 4.38	4.90	91.88 ± 3.56	3.87
	750	97.72 ± 1.42	1.46	97.01 ± 1.21	1.25
	8,000	95.42 ± 1.45	1.53	98.13 ± 0.29	0.30
Heart	30	93.48 ± 0.09	0.10	99.67 ± 4.42	4.44
	300	99.24 ± 2.78	2.81	91.80 ± 2.93	3.20
	3,000	92.46 ± 3.34	3.62	94.49 ± 4.64	4.91
Liver	30	87.83 ± 3.76	4.29	86.96 ± 8.73	10.05
	300	90.43 ± 3.11	3.44	89.61 ± 2.66	2.97
	3,000	89.26 ± 4.38	3.78	92.22 ± 3.31	3.59
Spleen	30	91.71 ± 3.54	3.87	93.20 ± 0.13	0.14
	300	93.19 ± 4.92	5.28	97.18 ± 5.52	5.69
	3,000	93.95 ± 2.63	2.81	96.17 ± 4.14	4.31
Lung	30	89.97 ± 5.38	5.98	95.38 ± 4.40	4.62
	300	90.73 ± 2.05	2.26	94.29 ± 1.20	1.28
	3,000	91.53 ± 3.41	3.73	88.37 ± 4.50	5.10
Kidney	30	94.14 ± 1.50	1.60	93.07 ± 2.35	2.53
	300	95.65 ± 7.14	7.47	96.99 ± 5.69	5.87
	3,000	97.52 ± 3.93	4.03	96.33 ± 3.06	3.18
Brain	30	95.85 ± 3.51	3.67	93.42 ± 3.00	3.22
	300	99.35 ± 8.05	8.11	93.21 ± 1.13	1.22
	3,000	94.48 ± 2.52	2.67	94.73 ± 4.66	4.92
Testis	30	94.31 ± 2.36	2.51	96.96 ± 2.27	2.35
	300	93.40 ± 1.72	1.85	92.86 ± 2.70	3.00
	3,000	95.38 ± 4.40	4.62	97.61 ± 142	1.46
Ovary	30	95.61 ± 5.12	5.36	99.35 ± 2.73	2.75
	300	94.73 ± 1.09	1.16	92.10 ± 1.11	1.21
	3,000	96.53 ± 5.48	5.68	92.18 ± 3.14	3.42
Muscle	30	96.16 ± 4.44	4.62	94.46 ± 2.72	2.89
	300	95.17 ± 4.49	4.72	91.64 ± 3.68	4.02
	3,000	96.84 ± 3.31	3.42	90.06 ± 5.10	5.67

#### 3.1.5 Stability

The stability of DF was assessed by comparing the concentrations of DF at three QC levels under different conditions with those of newly prepared samples of the same concentrations (Table 3). DF remained stable in the autosampler at 37°C for 4 h, at room temperature (25°C) for 24 h, after three freeze-thaw cycles, and in long-term storage at −80°C for 14 days. The results illustrated that there was no noteworthy deviation in DF under the above storage conditions.

**TABLE 3:** The stability of DF in rat plasma and tissues homogenate under different storage condition (*n* = 5).

Biological samples	Spiked concentration	Bench-top stability		Short-term stability		Freeze-thaw stability		Long-term stability	
		(25°C, 24 h)		(37°C, 4 h)		(Three cycles)		(−80°C, 14 days)	
	Con.(ng/ml)	Bias (%)	RSD (%)	Bias (%)	RSD (%)	Bias (%)	RSD (%)	Bias (%)	RSD (%)
Plasma	3	4.68	4.23	4.22	4.01	5.43	5.31	3.01	2.79
	750	−3.57	2.04	3.25	3.56	−3.76	1.94	−6.56	5.03
	8,000	1.04	3.20	−5.12	2.93	−1.28	0.89	1.97	2.31
Heart	30	−4.78	3.83	−6.16	5.34	−3.54	2.59	3.29	2.68
	300	−4.52	1.99	−3.21	4.22	2.88	3.01	−5.02	4.35
	3,000	−4.02	2.86	−3.89	4.13	3.62	3.47	−4.33	4.54
Liver	30	3.75	3.05	−1.96	1.68	−4.03	4.11	−5.20	3.85
	300	−4.01	4.04	−4.03	3.40	−2.82	1.55	−3.54	2.93
	3,000	2.80	1.21	−3.62	3.88	−5.70	2.89	−6.89	4.33
Spleen	30	2.79	2.32	−6.94	5.79	−3.33	3.47	−5.23	4.91
	300	−4.84	1.33	−5.23	5.48	2.05	2.66	−4.72	5.10
	3,000	3.02	0.98	−3.79	2.01	−3.21	3.45	−3.09	3.14
Lung	30	−4.59	3.54	−5.01	3.28	1.92	1.37	5.40	4.66
	300	−4.98	2.30	−4.87	1.23	−5.83	5.46	−6.84	5.36
	3,000	−3.05	1.08	2.98	1.36	−3.94	4.14	−4.32	3.70
Kidney	30	−3.97	2.10	0.98	1.29	−3.49	1.42	−4.50	3.86
	300	−3.19	1.95	−2.02	2.44	−4.69	2.56	4.07	3.75
	3,000	−4.01	2.05	2.65	2.38	−5.02	4.88	3.90	4.02
Brain	30	3.69	1.59	−3.29	4.59	−4.05	3.76	−3.39	1.92
	300	−3.54	2.02	−4.05	3.24	−4.33	2.31	−4.33	2.41
	3,000	−3.01	2.39	−1.09	1.36	3.89	1.78	−5.06	1.34
Testis	30	1.50	2.31	2.32	3.54	−2.79	2.27	−4.21	2.03
	300	−4.99	3.29	−3.29	3.05	−4.39	2.77	−4.64	3.65
	3,000	−3.52	1.25	3.01	2.60	−3.75	2.91	−5.30	4.90
Ovary	30	2.40	3.01	−4.12	3.59	−2.64	2.43	4.79	5.01
	300	1.69	1.88	−4.38	3.01	2.98	3.25	−4.30	3.88
	3,000	−4.03	2.01	−3.04	2.31	−5.77	4.53	3.83	3.91
Muscle	30	−3.85	3.12	−6.02	5.29	−6.03	3.78	7.01	3.49
	300	2.29	2.43	−3.72	3.84	−3.52	4.11	−4.44	4.17
	3,000	−3.20	1.03	1.03	2.03	−4.94	5.20	−4.32	3.44

### 3.2 Pharmacokinetics Study

**FIGURE 2:**
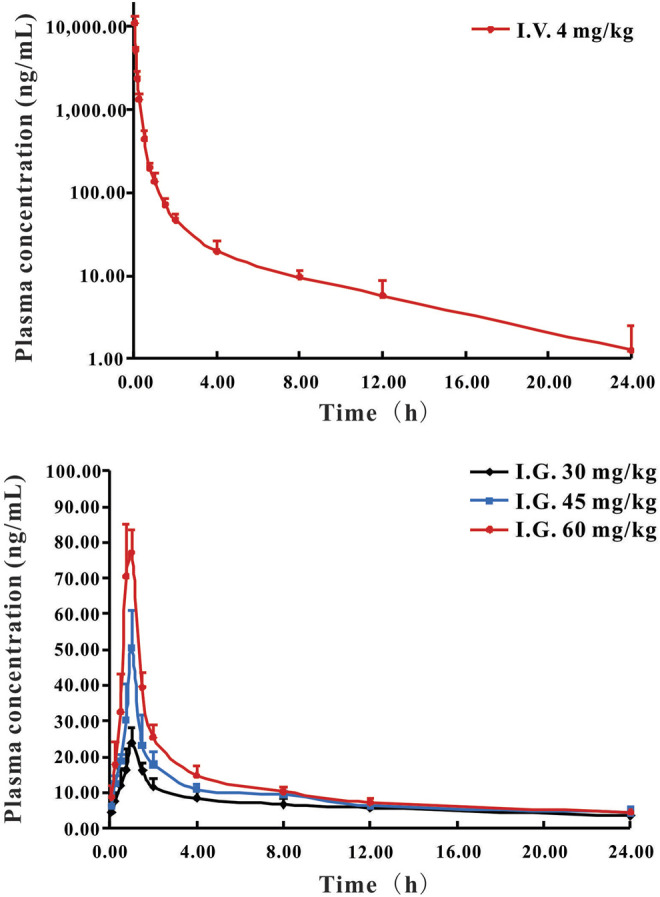
Mean plasma concentration-time curves of DF in rats following *i.v.* administration **(A)** at 4 mg/kg and *i.g.* administration **(B)** at a single dose of 30, 45, and 60 mg/kg DF to rats (Mean ± SD, *n* = 6).

The validated HPLC-MS/MS method was further applied to determine the pharmacokinetic behavior of DF in rats. The mean plasma concentration-time curves following a single *i.v.* administration of 4 mg/kg and a single *i.g.* administration of 30, 45, or 60 mg/kg are presented in Figures 2A,B, respectively.

The pharmacokinetic parameters of DF, calculated using non-compartmental analysis, are summarized in Table 4. For the *i.v.* administration, the plasma concentration of DF was rapidly decreased and cleared out from the plasma with the T_1/2_ at 2.81 h. DF was widely distributed with a large apparent distribution volume (1.56 L/kg) and extensive clearance (0.38 L/h/kg). For the *i.g.* administration, DF was rapidly absorbed into the circulatory system within 0.85 h, and the elimination rate was faster than that of the *i.v.* administration. The C_max_ and AUC_0−t_ of DF at the three dosages (30, 45, and 60 mg/kg) indicated an apparent dose-proportionality. There were no significant differences in the other parameters, including V_d_, T_1/2,_ and MRT_0-∞,_ among the three dosages. The mean oral bioavailability (F) of DF in the rats was 0.92 ± 0.08%.

**TABLE 4:** Non-compartmental pharmacokinetic parameters of DF after single *i.g.* and *i.v.* administration in rats (Mean ± SD, *n* = 6).

PK parameters	*i.g.* administration			*i.v.* administration
	30 mg/kg	45 mg/kg	60 mg/kg	4 mg/kg
T_1/2_ (h)	2.71 ± 0.67	3.19 ± 0.86	2.62 ± 0.14	2.81 ± 0.25
T_max_ (h)	0.85 ± 0.14	0.80 ± 0.11	0.85 ± 0.14	—
C_max_ (ng/ml)	28.98 ± 6.32	55.81 ± 9.60	95.33 ± 11.78	—
AUC_0-t_ (h·ng/ml)	163.84 ± 33.37	214.12 ± 15.29	283.11 ± 33.79	2,159.38 ± 392.02
AUC_0-∞_ (h·ng/ml)	176.49 ± 32.52	235.67 ± 19.54	300.20 ± 34.87	2,173.96 ± 399.99
V_d_ (L/kg)	697.10 ± 264.36	585.74 ± 152.70	761.09 ± 83.99	1.56 ± 0.45
Cl (L/h/kg)	174.34 ± 30.10	127.99 ± 10.55	201.91 ± 22.08	0.38 ± 0.09
MRT_0-t_ (h)	8.56 ± 0.22	8.21 ± 0.87	7.03 ± 0.24	0.69 ± 0.18
MRT_0-∞_ (h)	10.01 ± 0.68	10.06 ± 1.70	8.21 ± 0.35	0.80 ± 0.16
F (%)	0.92 ± 0.08			—

### 3.3 Tissue Distribution of DF in Rats

**FIGURE 3:**
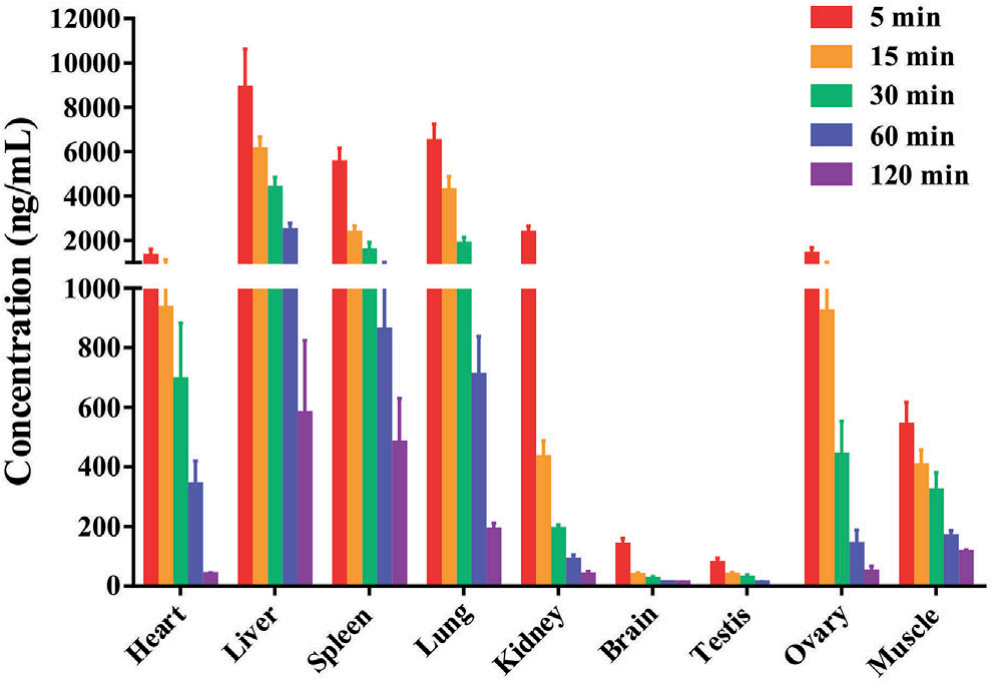
The mean concentrations of DF in the rat tissues were measured at 0.083, 0.25, 0.5, 1 and 2 h after rats received a single intravenous dose of the compound at 4 mg/kg (Mean ± SD, *n* = 6), DF primarily accumulated in the liver, lung, spleen and kidney.

The tissue distribution of DF was investigated following a single *i.v.* administration of 4 mg/kg in rats. The mean concentrations of DF in each tissue are presented in Figure 3. These data indicated that DF underwent a rapid and widespread distribution with no long-term accumulation in any of the tissues tested, consistent with the results of the PK study (Chi et al., 2019). After intravenous injection, all analytes presented with higher concentrations in the liver, lungs, spleens, kidneys, and heart, which might have been attributed to the high blood flow in these organs. The mainly distribution of DF in rats, which agrees fairly with previous anti-cancer reported in the literature (Menezes and Diederich, 2021; Wang et al., 2021). In addition, only a small amount of DF was detected in the brain, indicating that it may not easily cross the blood-brain barrier.

### 3.4 Plasma Protein Binding and Blood-Plasma Partitioning (BP Ratio)

The unbound fractions of DF in the rat plasma were 0.61 ± 0.35%, 5.05 ± 1.87%, and 6.11 ± 1.05% at concentrations of 50, 500, and 5,000 ng/ml, respectively. The BP ratios of DF were 1.11 ± 0.08 and 1.33 ± 0.13 for concentrations of 100 and 1,000 ng/ml, respectively.

### 3.5 Binding Studies on HSA

#### 3.5.1 UV Absorption Spectra of DF-HSA Complex

**FIGURE 4:**
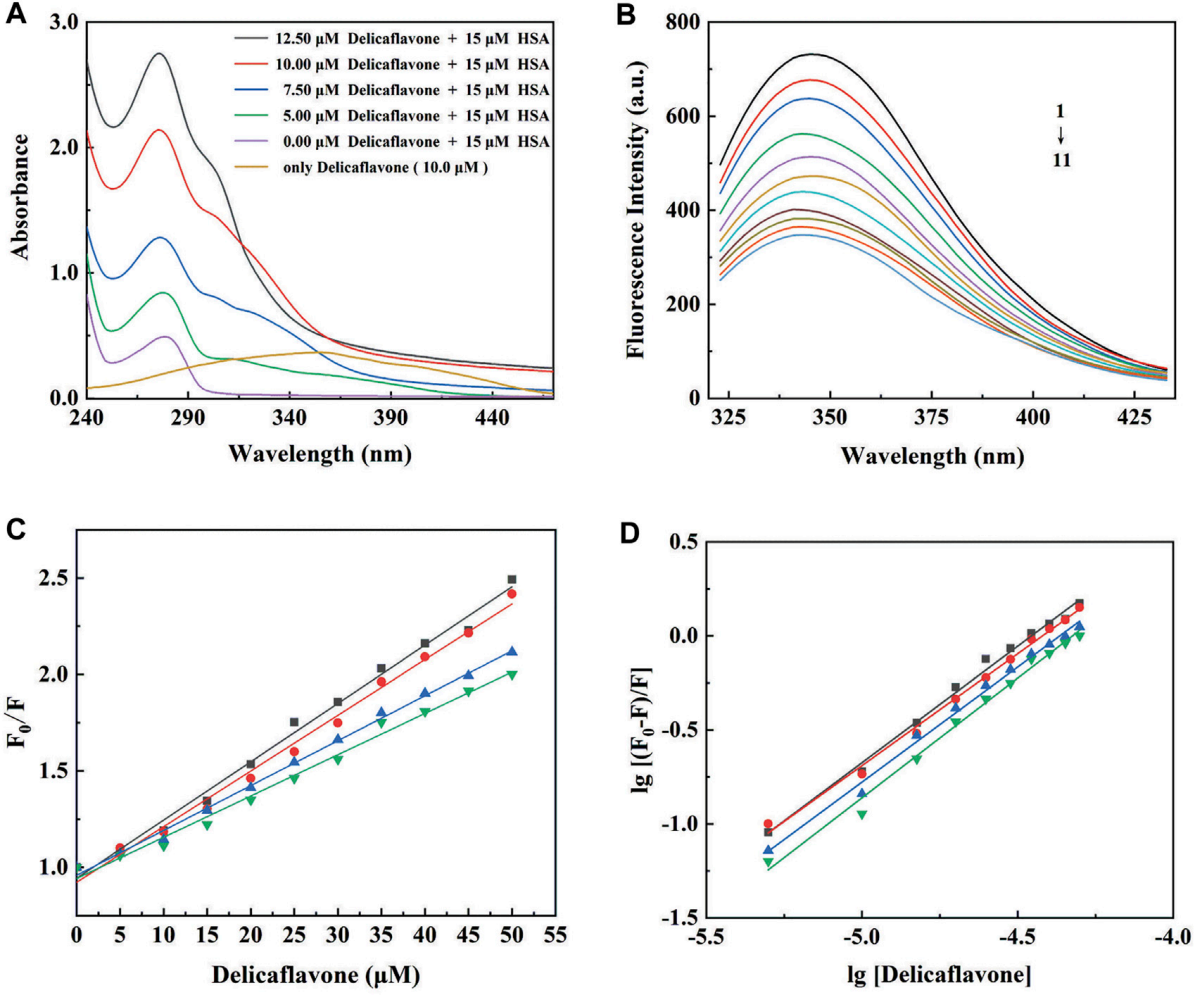
**(A)** UV-visible absorption spectra of DF (0, 5, 7.5, 10, and 12.5 μM) in the absence (purple line) or in the presence of HSA (15 μM) at pH 7.4; **(B)** The intrinsic fluorescence spectra of HSA (8 μM) in the absence (1) and presence of 5 (2), 10 (3), 15 (4), 20 (5), 25 (6), 30 (7), 35 (8), 40 (9), 45 (10), and 50 (11) μM DF; **(C)** The Stern-Volmer plots and **(D)** the modified Stern-Volmer plots for fluorescence quenching of HSA (8 μM) by DF at 298 (■), 303 (●), 308 (▲), and 313 (▼) K.

UV-visible spectroscopy provides the first evidence of DF-HSA interactions, which was slightly red-shifted at the maximum peak position. As shown in Figure 4A, the spectrum of DF in physiological saline (pH = 7.4, 1% methanol solution of final volume) is characterized by absorption bands with shoulders at 275 nm.

#### 3.5.2 Fluorescence Characterization of DF-HSA Complex

The indole moieties of the tryptophan residues of HSA make the proteins have a well-defined fluorescence emission spectrum, with a maximum at 350 nm (Rabbani and Ahn, 2019). DF was almost non-fluorescent under the present experimental conditions. The fluorescence intensity of HSA in the absence and presence of different concentrations of DF is shown in Figure 4B. As presented, the fluorescence intensity of HSA decreased regularly with increasing DF concentration, suggesting an increased hydrophobicity of the region surrounding the tryptophan amino acid residue (Tang et al., 2016).

#### 3.5.3 Fluorescence Quenching Mechanism

To elucidate the quenching mechanism of HSA by DF, temperature-dependent fluorescence quenching experiments were carried out at different temperatures, and the results are shown in Figure 4C and Supplementary Table S1. The K_SV_ values obtained at 298, 303, 308, and 313 K were 28.60 (±0.56) × 10^3^, 26.65 (±0.63) × 10^3^, 22.10 (±0.33) × 10^3^, and 19.75 (±0.49) × 10^3^, respectively. The Stern–Volmer plots for the HSA and DF systems were linear, with an inverse relationship between the K_SV_ values and the temperatures. According to the obtained results, the most probable quenching mechanism of HSA by DF might be static, and raising the temperature results in decreased stability of the DF-HSA complex.

#### 3.5.4 Analysis of the Binding Equilibrium, Thermodynamics, and Acting Forces

The values of the association constant (K_b_) and the number of binding sites (n) for the DF-HSA complex at different temperatures are shown in Figure 4D and are listed in Supplementary Table S2. The observed binding constants were nearly 10^5^ M^−1^, demonstrating the strong binding of DF to HSA at the studied temperatures (Zhang et al., 2019; Carvalho et al., 2020). It was also found that K_b_ decreased significantly with an increase in temperature, resulting in reduced stability of the DF-HSA complex at higher temperatures. The values of n are on the order of 1, which indicates that there is a single class-binding site for DF in the neighborhood of the tryptophan residue and that no more binding can occur even at higher DF concentrations (Bijari et al., 2015).

In general, the binding of ligands to macromolecules involves hydrogen bonding, van der Waals, and electrostatic interactions (Barreca et al., 2017). The van’t Hoff plot for the evaluation of the thermodynamic parameters of the DF-HSA interaction is shown in Supplementary Table S3. The high negative ΔG° value (nearly ΔG° = −31 KJ mol^−1^) is indicative of a spontaneous process during the binding of DF to HSA. Meanwhile, the high negative ΔH° value, accompanied by a negative entropy change (ΔS°), suggests that the binding process is mainly enthalpy driven. Therefore, both hydrogen bonds and van der Waals interactions play a significant role in the binding of DF to HSA, according to the above-mentioned data (Cao et al., 2019).

### 3.6 Molecular Docking of DF With HSA

**FIGURE 5:**
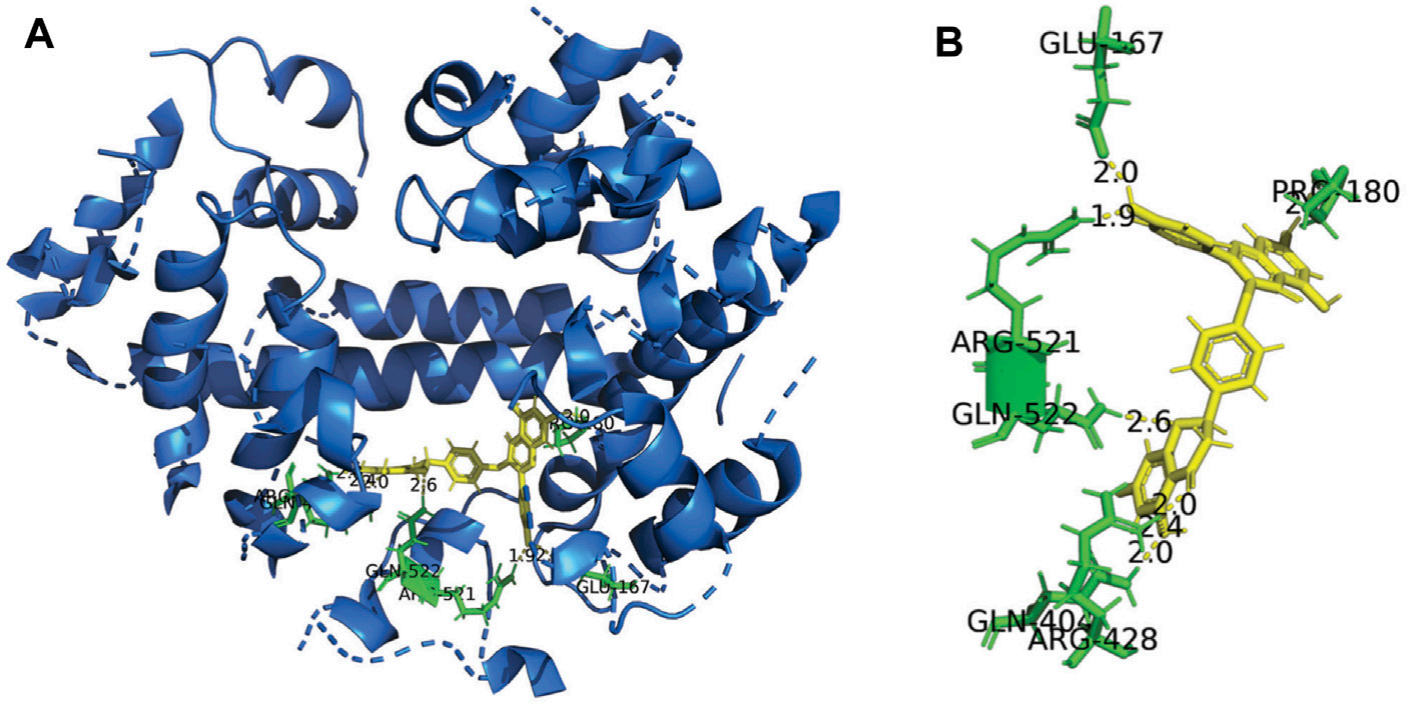
Three-dimensional structure of HSA Complexed with DF. **(A)** The HSA structure has been rendered with ribbons and line, while DF is rendered as tune. The inset shows a magnification of the binding site with DF represented using stick model; **(B)** Representation of the amino acid residues in the binding site with their hydrogen bonds and van der Waals radii.

In the present study, the Sybyl-x 1.3 program was used to characterise the possible binding site of DF on HSA. The 3D crystal structure and the 2D schematic of DF with its best docking state in the active site of HSA are depicted in Figure 5A, with a binding energy of −5.4 kcal mol^−1^. The polar value and total score of the DF-HSA complex were 5.72 and 6.52, respectively. Targets of ligands with high total scores are usually calculated to have a correspondingly low binding free energy. For the docking of HSA, Figure 5B shows the DF stabilized by the seven hydrogen bonds involving the amino acid residues Glu167, Pro180, Arg428, Gln404, Arg521, and Gln522 in HSA. According to this result, the interaction between DF and HSA is not exclusively through hydrogen bonds, but also involves ionic and polar interactions. These interactions play an important role in stabilizing the DF-HSA complex, which blocks light, a result that is in accordance with the results that provide experimental evidence to explain the fluorescence quenching of HSA emission and the absorbance decrease at 350 nm.

## 4 Discussion

Many studies have demonstrated that DF possesses favorable anti-tumor potential and minimal side effects, and deserves to be further development as an anti-tumor candidate (Sui et al., 2017; Yao et al., 2019). In the preclinical phase, pharmacokinetic studies are a prerequisite to guarantee that the tested drugs have appropriate drug-like properties. Understanding pharmacokinetic properties is crucial for lead identification and optimization in drug discovery and preclinical development, as well as for dosage regimen design and drug formulation developed.

In the pharmacokinetic curves after a single *i.v.* administration and *i.g.* administration in rats, DF was rapidly absorbed and eliminated from rat plasma, with an average T_1/2_ (2.81 h, 2.84 h). The mean absolute bioavailability of DF in rats was estimated to be 0.98% after oral administration three doses. These results correlate with our previous study on total bioflavonoid extracts from *S. doederleinii* with inferior bioavailability, which suggested that biflavonoids have poor solubility and permeability (Shen et al., 2021). In addition, the linear pharmacokinetic behavior of DF was observed across the investigated dosage range (30–60 mg/kg). Delicaflavone was widely distributed, with a large apparent distribution volume (585.74, 697.10, and 761.09 L/kg) and extensive clearance (127.99, 173.34, and 201.91 L/h/kg) at three oral doses, respectively. It was inferred that the rapid and extensive biotransformation of DF in rats might be the main cause of the low bioavailability of DF after oral administration (Wang et al., 2014; Zhong et al., 2020).

The tissue distribution results suggest that DF exhibits rapid and wide distribution to all of the examined tissues and organs throughout the body following intravenous administration of DF (4 mg/kg) to rats, which would conceivably imply a rapid pharmacological effect (Lv et al., 2019). In our analysis of the tissue distribution, concentrations of DF may reach to the highest level before 0.08 h in some tissues with high blood flow. Compared with plasma exposure, extremely low levels of DF were detected in brain, indicating that it cannot effectively cross the blood-brain barrier.

The drug-drug interaction (DDI), pharmacodynamics, and pharmacokinetic properties of a drug are closely related to its reversible binding to plasma or serum proteins. The results of the plasma protein binding studies were consistent at the concentrations of 50, 500, and 5,000 ng/ml, with unbound fraction values of 0.61, 5.05, and 6.11%, respectively. Delicaflavone displayed a high affinity for plasma/serum protein, which is consistent with its high lipophilicity, and the rate of plasma protein binding was nonlinear concentration-dependent (Nation et al., 2018; Chen et al., 2020).

Multi-technique spectroscopic approaches and molecular docking were used to elucidate the nature of the interaction between DF and HSA. UV-visible spectroscopy provided the first evidence of DF-HSA interaction, which was slightly red-shifted at the maximum peak position. In the present study, the binding constant of DF to HSA was determined by the intrinsic fluorescence quenching of the aromatic amino acids of HSA. The obtained binding constants of the DF-HSA complex formation were calculated to be on the order of 10^5^ M^−1^, which was found to be within the range reported for HSA binding constants (10^3^∼10^8^ M^−1^) (Carvalho et al., 2020). In addition, the Gibbs free energy associated with the binding at 298, 303, 308, and 313 K was found to be negative (nearly ΔG° = −31 KJ mol^−1^). These results indicate that the strong and spontaneous binding of DF to HSA is responsible for poor pharmacokinetic profiles and is a limiting therapeutic step, or that nanocarriers could be designed to fine-tune lipophilicity, plasma protein binding, and facilitate diffusion across the membrane (Valic et al., 2020).

## 5 Conclusion

To the best of our knowledge, this is the first comprehensive pharmacokinetic and tissue distribution study of DF in rats and its binding properties to HSA. Various spectroscopic techniques, as well as *in silico* methods, have indicated minor changes in the HSA conformational structure in interactions with DF. A ground-state complex formation between DF and HSA was found, and thermodynamic analysis of the binding parameters revealed that hydrogen bonds and van der Waals interactions are the major forces involved in the binding interaction. A sensitive, rapid, and robust HPLC-MS/MS method was used to detect DF in the rat plasma and tissues (Bijari et al., 2015). The absolute bioavailability of DF in rats was very low because the drug is highly bound to plasma proteins in the range of 93.88–99.38% and is a limiting therapeutic step. Alternatively, it suggests that nanocarriers could be designed to fine-tune lipophilicity, plasma protein binding, and facilitate diffusion across the membrane (Zhang et al., 2019). The present pharmacokinetic and biophysical interaction study on DF will provide helpful information for further investigation and the development of DF, as well as for optimizing the properties of drugs and nanocarriers that are able to cope with the pharmacokinetic challenges of anticancer therapeutics.

## Data Availability

The original contributions presented in the study are included in the article/Supplementary Material, further inquiries can be directed to the corresponding authors.
