# Supplementary material for: Pharmacokinetics, Tissue Distribution, and Human Serum Albumin Binding Properties of Delicaflavone, a Novel Anti-Tumor Candidate
Source: Front Pharmacol. 2021 Nov 17;12:761884. doi: 10.3389/fphar.2021.761884 (PMC8635734; doi:10.3389/fphar.2021.761884)
Supplement: Supplementary file 1 [file DataSheet2.docx]

**Supplementary Material**

**Pharmacokinetics, Tissue Distribution, and Human Serum Albumin Binding Properties of** **Delicaflavone, a Novel Anti-tumor Candidate**

**Bing Chen^1,2†^, Hongbin Luo^2,3†^, Weiying Chen^2,4^, Qishu Huang^2^, Kaifan Zheng^2^, Dafen Xu^2^, Shaoguang Li^2^, Ailin Liu^2^, Liying Huang^2^, Yanjie Zheng^2*^, Xinhua Lin^1,2*^ and Hong Yao^2,5*^**

^1^Key Laboratory of Nanomedical Technology (Education Department of Fujian Province), School of Pharmacy, Nano Medical Technology Research Institute, Fujian Medical University, Fuzhou, China

^2^Department of Pharmaceutical Analysis, School of Pharmacy, Fujian Medical University, Fuzhou, China

^3^Department of Orthopedic, The First Affiliated Hospital, Fujian Medical University, Fuzhou, China

^4^Department of Pharmacy, Xiamen Humanity Hospital, Fujian Medical University, Xiamen, China

^5^Fujian Key Laboratory of Drug Target Discovery and Structural and Functional Research, Fujian Medical University, Fuzhou, China

*** Correspondence:**

Hong Yao

yauhung@126.com

Xinhua Lin

13906909638@163.com;

Yanjie Zheng

gillzheng@fjmu.edu.cn

**^†^**These authors have contributed equally to this work and share first authorship

**List of Supplementary Material Captions**

**TABLE S1∣**The Stern-Volmer quenching constant (K_SV_) and quenching rate constant (kq) for binding of DF to HSA at different temperatures (Mean ± SD, n=3).

**TABLE S2∣**The association constant (K_b_) and number of binding site (n) for binding of DF to HSA at different temperatures (Mean ± SD, n=3).

**TABLE S3∣**Thermodynamic parameters for binding of DF to HSA (Mean ± SD, n=3).

**TABLE S1∣**The Stern-Volmer quenching constant (K_SV_) and quenching rate constant (kq) for binding of DF to HSA at different temperatures (Mean ± SD, n=3).

| **Complex** | **T (K)** | **K_SV_ × 10^3^ (M^-1^)** | **K_q_ × 10^11^ (M^-1^ S^-1^)** | **R^2^** |
| --- | --- | --- | --- | --- |
| DF-HSA | 298 | 28.60 ± 0.56 | 28.60 ± 0.56 | 0.9991 |
|  | 303 | 26.65 ± 0.63 | 26.65 ± 0.63 | 0.9988 |
|  | 308 | 22.10 ± 0.33 | 22.10 ± 0.33 | 0.9996 |
|  | 313 | 19.75 ± 0.49 | 19.75 ± 0.49 | 0.9991 |

R^2^ is the correlation coefficient for the K_SV_ values.

**TABLE S2∣**The association constant (K_b_) and number of binding site (n) for binding of DF to HSA at different temperatures (Mean ± SD, n=3).

| **Complex** | **T (K)** | **K_b_ × 10^5^ (M^-1^)** | **n** | **R^2^** |
| --- | --- | --- | --- | --- |
| DF-HSA | 298 | 3.304 | 1.238 | 0.9934 |
|  | 303 | 3.199 | 1.190 | 0.9947 |
|  | 308 | 2.192 | 1.224 | 0.9939 |
|  | 313 | 1.815 | 1.273 | 0.9910 |

R^2^ is the correlation coefficient for the K_b_ values.

**TABLE S3∣**Thermodynamic parameters for binding of DF to HSA (Mean ± SD, n=3).

| **Complex** | **T (K)** | **ΔS^o^ (J mol^-1^ K^-1^)** | **ΔH^o^ (kJ mol^-1^)** | | **ΔG^o^ (kJ mol^-1^)** |
| --- | --- | --- | --- | --- | --- |
| DF-HSA | 298 | -6.831 | | -33.69 | -31.65 |
|  | 303 |  |  |  | -31.62 |
|  | 308 |  |  |  | -31.58 |
|  | 313 |  |  |  | -31.55 |
