# Supplementary material for: Pharmacokinetics, Tissue Distribution, and Human Serum Albumin Binding Properties of Delicaflavone, a Novel Anti-Tumor Candidate
Source: Front Pharmacol. 2021 Nov 17;12:761884. doi: 10.3389/fphar.2021.761884 (PMC8635734; doi:10.3389/fphar.2021.761884)
Supplement: Supplementary file 2 [file DataSheet1.docx]

**Supplementary Material**

**Pharmacokinetics, Tissue Distribution, and Human Serum Albumin Binding Properties of** **Delicaflavone, a Novel Anti-tumor Candidate**

**Bing Chen^1,2†^, Hongbin Luo^2,3†^, Weiying Chen^2,4^, Qishu Huang^2^, Kaifan Zheng^2^, Dafen Xu^2^, Shaoguang Li^2^, Ailin Liu^2^, Liying Huang^2^, Yanjie Zheng^2*^, Xinhua Lin^1,2*^ and Hong Yao^2,5*^**

^1^Key Laboratory of Nanomedical Technology (Education Department of Fujian Province), School of Pharmacy, Nano Medical Technology Research Institute, Fujian Medical University, Fuzhou, China

^2^Department of Pharmaceutical Analysis, School of Pharmacy, Fujian Medical University, Fuzhou, China

^3^Department of Orthopedic, The First Affiliated Hospital, Fujian Medical University, Fuzhou, China

^4^Department of Pharmacy, Xiamen Humanity Hospital, Fujian Medical University, Xiamen, China

^5^Fujian Key Laboratory of Drug Target Discovery and Structural and Functional Research, Fujian Medical University, Fuzhou, China

*** Correspondence:**

Hong Yao

yauhung@126.com

Xinhua Lin

13906909638@163.com;

Yanjie Zheng

gillzheng@fjmu.edu.cn

**^†^**These authors have contributed equally to this work and share first authorship

**List of Supplementary Material Captions**

**FIGURE S1∣**Representative chromatograms of IS (100 ng/mL) and DF in (A) Heart, (B) Liver, (C) Spleen, (D) Lung, (E) Kidney, (F) Brain, (G) Testis, (H) Ovary, and (I) Muscle for tissue distribution study. (a) Biological sample collected from the rat 5 min after *i.v.* administration of DF at a dose of 4 mg/kg; (b) Blank plasma spiked with DF and IS; (c) Blank biological sample.

**
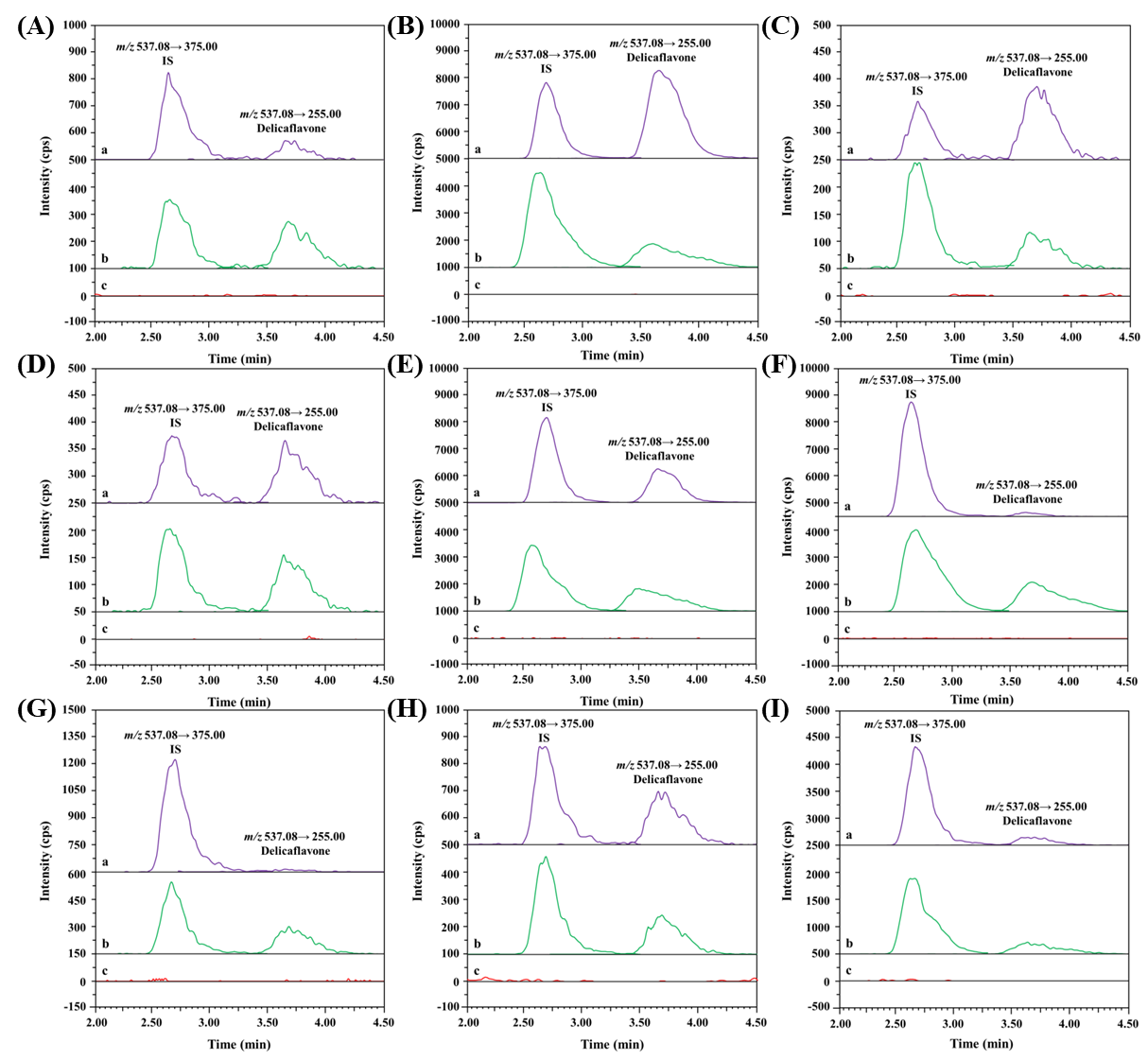
**

**FIGURE S1∣**Representative chromatograms of IS (100 ng/mL) and DF in (A) Heart, (B) Liver, (C) Spleen, (D) Lung, (E) Kidney, (F) Brain, (G) Testis, (H) Ovary, and (I) Muscle for tissue distribution study. (a) Biological sample collected from the rat 5 min after *i.v.* administration of DF at a dose of 4 mg/kg; (b) Blank plasma spiked with DF and IS; (c) Blank biological sample.
